# Hematopoietic stem cell transplantation and quality of life during the first year of treatment

**DOI:** 10.1590/1518-8345.2474.3065

**Published:** 2018-10-25

**Authors:** Angela da Costa Barcellos Marques, Ana Paula Szczepanik, Celina Angélica Mattos Machado, Pâmella Naiana Dias Santos, Paulo Ricardo Bittencourt Guimarães, Luciana Puchalski Kalinke

**Affiliations:** 1 Universidade Federal do Paraná, Complexo Hospital de Clínicas, Curitiba, PR, Brazil.; 2 Hospital Vita Curitiba, Supervisão de Enfermagem, Curitiba, PR, Brazil.; 3 Universidade Federal do Paraná, Departamento de Estatística, Curitiba, PR, Brazil.; 4 Universidade Federal do Paraná, Departamento de Enfermagem, Curitiba, PR, Brazil.

**Keywords:** Quality of Life, Hematopoietic Stem Cell Transplantation, Bone Marrow Transplantation, Hematologic Neoplasms, Oncology Nursing, Drug Therapy

## Abstract

**Objective:**

to evaluate the quality of life of adult patients with hematological cancer comparing Hematopoietic Stem Cell Transplantation modalities during the first year of treatment.

**Method:**

this is an observational and longitudinal research with 55 participants. Data collection was performed in six steps: before transplantation, pancytopenia, before hospital discharge, after 100, 180 and 360 days, in a reference hospital in Brazil for this treatment. The international instruments Quality of Life Questionnaire - Core 30 and Functional Assessment Cancer Therapy - Bone Marrow Transplantation were validated and translated into Portuguese (Brazil).

**Results:**

the mean age of participants was 36 years, 65% (n = 36) had leukemia diagnosis and 71% (n = 39) had undergone allogenic transplantation. In the Quality of Life Questionnarie - Core30 instrument, the pain symptom was significant between the first and second stages, and loss of appetite between the third and fourth stages, both in the allogenic group. In the Functional Assessment Cancer Therapy - Bone Marrow Transplantation, the functional well-being domain was significant between the third and fourth stages, also in the allogenic group.

**Conclusions:**

although the aggressiveness of treatment affects quality of life, patients consider it satisfactory after the first year. There are few significant differences between autologous and allogenic patients, and both groups have recovered in the course of the process.

## Introduction

Some malignant and non-malignant diseases can be treated with hematopoietic stem cell transplantation (HSCT). Since it is a complex and aggressive procedure, it demands demand specific care with professionals from different areas inserted in the same therapeutic context. The treatment is relatively long and involves risks that predispose the patient to a broad spectrum of complications that need to be managed in order not to threaten his/her life or affect his/her survival and quality of life (QoL). 

The hematological cancers are among the malignancies, with considerable incidence in Brazil and in the world. According to the National Cancer Institute José Alencar Gomes da Silva (INCA), estimates for the biennium 2016/2017 indicate the occurrence of 22,780 cases, 12,210 among men and 10,570 among women^1^. For the International Agency for Research on Cancer (IARC), from the World Health Organization (WHO), 948,942 cases are estimated in 2020^2^.

Cancer has the potential to negatively impact patients’ QoL, as it creates suffering because of uncertainty about the future and how their body will react to treatment^3^. It entails physical, psychological and emotional changes, with consequent loss of self-esteem interfering in patients’ survival and QoL^4^ ^-^ ^5^.

In recent decades, QoL has been the focus of studies, mainly in the area of oncology. The development and technological improvement of the therapeutics for hematological cancer has increased the possibility of prolonging the life of patients, reflecting in a greater attention to the QoL, that started to be as important as the survival^6^ ^)^ and aroused the interest of researchers in knowing how patients’ lives are affected by the diseases^7^ ^-^ ^8^.

There are varied definitions and conceptions for the term “Quality of Life”. Its concept is broad and multidimensional, with social, health or economic parameters^9^ ^-^ ^10^. The World Health Organization (WHO) defines it as individual’s perception of their position in life in the context of the culture and value systems in which they live in relation to their goals, expectations, standards and concerns”^11^. For this research, we decided to use the WHO definition because we understood that its concept is more comprehensive and possibly contemplates the entire therapeutic path that the patient with hematological cancer submitted to HSCT can go through.

Despite the complexity and aggressiveness of HSCT, its performance has increased every year. In 2016, 2,270 HSCTs were performed in Brazil by 43 transplantation centers. In the first quarter of 2017, there were 516 HSCTs, of which 316 were autologous and 200 allogenic^12^. Approximately 30,000 transplantations have been performed in our country since 1979^13^.

The modalities of HSCT are denominated as autologous or autogenic and allogenic. They are determined according to the type of donor of hematopoietic stem cells (HSCs). In autologous HSCT, the HTCs are collected from the patient him/herself before the conditioning phase; they are basically stored and reinfused later. In allogenic HSCT, the HTCs come from a donor that may be related or not to the patient^14^ ^-^ ^16^. 

Each HSCT modality has specificities, with pre-established protocols and chemotherapeutic regimens according to the disease, besides requiring care at different levels of complexity. The allogenic HSCT has some particularities in terms of variables to be controlled, as there is concern about the necessary compatibilities between donor and recipient. In addition, the fact that HSCs come from another person, relative or not, may increase the risk of complications, such as Graft versus Host Disease (GVHD), thus affecting multiple organ systems and implying changes in the domains of QoL^16^ ^-^ ^17^. 

However, both modalities have their own demands, and there are several factors, such as the existence of previous comorbidities associated or not with hematologic cancer, older age or not, diagnosis time, prognosis and even social conditions, among others that may interfere in the treatment, regardless of the type of transplantation performed.

During the therapeutic process, the patient undergoes some critical stages in which complications, besides putting their life at risk, can negatively affect their QoL, as symptoms that have a disabling potential may appear^16^ ^,^ ^18^. In addition to the physical complications, the patient may suffer with emotional and social changes during treatment. They may feel fear and anguish, and miss the family and friends, since social isolation is necessary in the early stages of treatment.

The QoL of patients worsens as the severity of the symptoms increases^19^. Knowing the specific changes in the QoL of the patient at each stage of the treatment enables the professionals involved in this context, especially the nurse, to establish an individualized and effective care plan, assisting the patient in facing their clinical condition, as well as aiming at a better survival. In this sense, it is relevant to carry out studies focused on this theme.

Thus, the objective of the present study was to evaluate the QoL of adult patients with hematological cancer submitted to HSCT in the different stages of treatment during the first year and to compare the autologous and allogenic transplantation modalities.

## Method

This research is part of the thematic project “Quality of Life Assessment of Patients with Hematologic Neoplasm Submitted to Hematopoietic Stem Cell Transplantation”, approved by the Research Ethics Committee of the Health Sciences Sector of the Federal University of Paraná under the opinion number 411,548 whose objective was to evaluate QoL of patients up to five years after HSCT.

The use of the instruments was authorized by the European Organization Research Treatment of Cancer (EORTC) and the Functional Assessment of Chronic Illness Therapy (FACIT), which made the questionnaires available via download directly to the researcher after registering the research project.

This is a longitudinal and observational research developed in the Bone Marrow Transplantation Service (STMO in Portuguese) of a federal teaching hospital in Curitiba, national reference in HSCT, from September 2013 to November 2016.

The non-probabilistic sample, however, was based on the number of patients attended in the years 2010 to 2012 plus 50%, due to the possibility of losses because of the treatment characteristics, was composed of 55 participants. Inclusion criteria were age equal to or greater than 18 years, diagnosis of hematologic cancer and having been submitted to HSCT. Participants who did not have the physical conditions to complete the instruments were excluded from the survey. Three patients were excluded due to loss of follow-up; 12 died before completing 100 days of HSCT, two before completing 180 days and six days before completing 360 days. 

Data were collected in the inpatient and outpatient wards of HSCT in six stages, namely before HSCT, pancytopenia period, before discharge, after 100 days, after 180 days, and after 360 days of HSCT. Sociodemographic and clinical data were collected with a specific instrument in pre-HSCT stage. In all stages, we applied the instruments Quality of Life Questionnaire-Core 30 (QLQ C-30) - version 3.0 Brazilian Portuguese, developed by the European Organization for Research and Treatment of Cancer (EORTC) and the Functional Assessment Cancer Therapy - Bone Marrow transplantation (FACT-BMT) - version 4.0 Brazilian Portuguese, prepared by the Functional Assessment of Chronic Illness Therapy (FACIT). The QLQ C-30 is divided into functional and symptom scales and its results are calculated according to the EORTC Scoring Manual^20^. The FACT-BMT is divided into domains and its results are calculated as described in the FACIT Scoring Manual^21^. 

Sociodemographic and clinical data were analyzed descriptively and expressed in absolute and relative frequency. The data of the QoL instruments were organized into tables and analyzed according to EORTC and FACIT guidelines, expressed as mean (M).

The Mann Whitney test was used to compare the types of transplantation and the Friedman test was applied for the comparison between the stages, complemented by the Test of Minimum Important Difference, for multiple comparisons (p-value), in which the level of significance of 5% for results with p-value below 0.05 were considered significant (p <0.05). The calculations were performed by a statistician. The Statistica 7.0 software was used for analysis.

## Results

The sociodemographic characterization of the sample showed that the mean age was 36 years, 53% (n = 29) were male, 55% (n = 30) were married or declared stable union. Regarding education, 44% (n = 24) declared having completed high school and 64% (n = 35) declared themselves to be economically active. Regarding the clinical characterization, 65% (n = 36) presented some type of leukemia and 71% (n = 39) had undergone allogenic HSCT.

The results expressed in Table 1 demonstrate the means obtained between the autologous and allogenic groups, measured by the QLQ-C30 in the six stages of the research. The functional scale shows that in both groups there was recovery of physical function and social function after 360 days of transplantation when compared to the pre-HSCT stage; in this scale, the higher the mean, the better the performance. In the symptom scale, the means demonstrate increased fatigue in the autologous group after 360 days in relation to the pre-HSCT stage; in this scale, the higher the mean, the more intense the symptomatology.

**Table 1:** Significant scores of the Quality of Life Questionnaire - Core 30 of the patients submitted to autologous and allogenic transplantation obtained in the six stages of the research. Curitiba, PR, Brazil, 2013-2016 (n=55)

**Quality of Life Questionnaire - Core 30**												
**SCORES**	Before HSTC* (n=55)		**Pancytopenia (n=50)**		**Before discharge** **(n=49)**		**After 100 days** **(n=41)**		**After 180 days** **(n=38)**		**After 360 days** **(n=32)**	
	**Means**		**Means**		**Means**		**Means**		**Means**		**Means**	
	**Aut** ^**†**^	**Alo** ^**‡**^	**Aut** ^**†**^	**Alo** ^**‡**^	**Aut** ^**†**^	**Alo** ^**‡**^	**Aut** ^**†**^	**Alo** ^**‡**^	**Aut** ^**†**^	**Alo** ^**‡**^	**Aut** ^**†**^	**Alo** ^**‡**^
**Overall QoL** ^**§**^	**70.8**	**79.2**	**59.3**	**55.3**	**73.9**	**66.7**	**80.7**	**71.4**	**75.6**	**77.5**	**72.7**	**70.6**
**Functional Scale**												
**Physical function**	**72.9**	**77**	**50.8**	**57.6**	**57.5**	**68.2**	**82.5**	**74.7**	**79.4**	**80.5**	**83.6**	**83.8**
**Personal Performance**	**79**	**79**	**50**	**41.1**	**52**	**61.6**	**82**	**72.6**	**88.2**	**77.5**	**90 .9**	**87.3**
**Social role**	**62.5**	**52.1**	**46.8**	**33.3**	**46.8**	**35.8**	**71.7**	**52.9**	**86.1**	**67.9**	**81.8**	**75.4**
**Scale Symptoms/Items**												
**Fatigue**	**22.2**	**21.3**	**47.9**	**55.8**	**35.4**	**45.4**	**26.5**	**28.9**	**27.7**	**24.7**	**29.8**	**22.2**
**Nausea and vomiting**	**8.3**	**9.4**	**43.7**	**47.5**	**35.4**	**35.8**	**6.4**	**13.1**	**8.3**	**5.7**	**7.5**	**11.1**
**Pain**	**30.2**	**14.1**	**38.5**	**68.1**	**18.7**	**28.7**	**20.5**	**17.2**	**18**	**13.4**	**25.7**	**12.7**
**Loss of appetite**	**20.8**	**16.2**	**56.2**	**68.6**	**47.9**	**61.6**	**5.1**	**27.3**	**16.6**	**16.6**	**6**	**12.7**
**Diarrhea**	**4.1**	**5.9**	**50**	**54.9**	**33.3**	**26.2**	**15.3**	**13.1**	**13.8**	**7.6**	**12.1**	**1.5**

*HSCT: Hematopoietic stem cell transplantation; † Aut: Autologous; ‡ Alo: Allogenic; §QoL: Quality of Life

Table 2 shows the means obtained by the group of patients submitted to autologous and allogenic HSCT, measured by FACT-BMT in the six stages of the study. It is observed that only in the pre-HSCT stage the allogeneic group obtained averages higher than the autologous group in all domains. However, in all other stages, the means of the allogeneic group were lower than those of the autologous group, but with no significant difference.

**Table 2:** Significant scores of the Functional Assessment of Cancer Therapy - Bone Marrow Transplantation of patients submitted to autologous and allogenic transplantation obtained in the six stages of the research. Curitiba, PR, Brazil, 2013-2016 (n=55)

Functional Assessment of Cancer Therapy Bone Marrow Transplantation												
Domain	Before HSCT* (n=55)		Pancytopenia (n=50)		Before discharge (n=49)		After 100 days (n=41)		After 180 days (n=38)		After 360 days (n=32)	
	Means		Means		Means		Means		Means		Means	
	Aut^†^	Alo^‡^	Aut^†^	Alo^‡^	Aut^†^	Alo^‡^	Aut^†^	Alo^‡^	Aut^†^	Alo^‡^	Aut^†^	Alo^‡^
Physical well-being	21	22.3	16.4	14.5	19.9	18.7	23	21.2	23.9	22.7	24.2	23.2
Functional well-being	18.9	19.5	15.1	14.6	16.9	15.4	20.2	16.2	18.6	18.6	19.9	18.2
Additional concerns	27.5	27.9	22.6	23	24.7	23.4	30.3	27.4	31.1	29	29.9	29.1
FACTG^§^	80.2	80.7	68.4	67.3	74.2	71.5	84.3	77.5	84.1	81.3	83.9	80.3
Overall QoL	107	108	91.4	90.3	98.9	95	114	105	115	110	113	109

*HSCT: Hematopoietic stem cell transplantation; †Aut: Autologous; ‡Alo: Allogenic; §FACTG: Overall assessment (physical well-being/social and family well-being/emotional well-being/functional well-being)

The means of overall QoL values measured by the QLQ-C30 and by the FACT-BMT, expressed in Tables 1 and 2, respectively, showed similar performance among patients submitted to autologous and allogenic HSCT in the six stages of the research.

The comparison between the modalities of HSCT, as demonstrated in Table 3, showed that in both the overall QoL measured by QLQ-C30 and in the general QoL measured by FACT-BMT, there was no statistically significant difference in any stage of the study. However, in relation to the symptom of pain and loss of appetite measured by the QLQ-C30, as well as the functional well-being evaluated with the FACT-BMT, there was a significant difference between autologous and allogenic transplantation in the pancytopenia period and after 100 days, respectively.

**Table 3:** Comparison between autologous and allogeneic transplantation measured by the Quality of Life Questionnaire - Core 30 questionnaire and the Functional Assessment of Cancer Therapy - Bone Marrow Transplantation in the six stages of the research. Curitiba, PR, Brazil, 2013-2016 (n=55)

Domain	Before HSCT* (n=55)	Pancytopenia (n=50)	Before discharge (n=49)	After 100 days (n=41)	After 180 days (n=38)	After 360 days (n=32)
	p-value	p-value	p-value	p-value	p-value	p-value
Quality of Life Questionnaire - Core 30						
Overall QoL^†^	0.094	0.571	0.266	0.298	0.841	0.785
Physical function	0.503	0.337	0.075	0.353	0.792	0.969
Personal Performance	0.993	0.251	0.428	0.249	0.312	0.611
Social role	0.213	0.099	0.223	0.186	0.207	0.639
Fatigue	0.905	0.369	0.325	0.836	0.525	0.289
Nausea and vomiting	0.847	0.750	0.924	0.151	0.841	0.815
Pain	0.052	0.004^‡^	0.285	0.480	0.359	0.180
Loss of appetite	0.734	0.297	0.240	0.031^‡^	0.938	0.755
Diarrhea	0.862	0.688	0.576	0.688	0.653	0.155
Functional Assessment of Cancer Therapy Bone Marrow Transplantation						
Physical well-being	0.250	0.278	0.620	0.382	0.792	0.852
Functional well-being	0.790	0.658	0.240	0.031^‡^	0.699	0.348
Additional concerns	0.491	0.734	0.428	0.151	0.327	0.884
FACTG^§^	0.804	0.297	0.506	0.186	0.676	0.574
Overall QoL^†^	0.666	0.688	0.506	0.205	0.545	0.519

Notes: Mann-Whitney test; *HSCT: Hematopoietic stem cell transplantation; †QoL: Quality of life; ‡Statistically significant data; §FACTG: Overall assessment (physical well-being/social and family well-being/emotional well-being/functional well-being)

**Figure 1:**
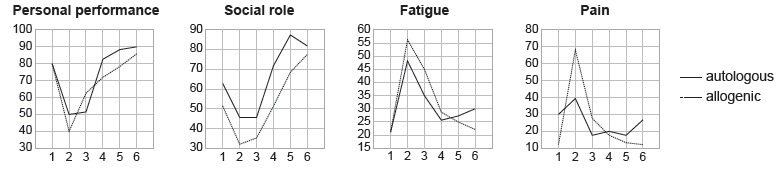
Comparison of the six stages of the research using the domains measured by Quality of Life Questionnaire - Core 30 (QLQ-C30) of patients submitted to autologous and allogenic HSCT. Curitiba, PR, Brazil, 2013-2016, (n=55)

Note: Friedman test

The comparison of the autologous and allogenic transplantations, expressed in Figure 1, of the domains measured by the QLQ-C30, shows that in the personal performance there is a significant reduction of the means in the periods of pancytopenia and before discharge in relation to the pre-HSCT stage, with gradual recovery up to 360 days. In the social function, there was a similar behavior of the means in both groups, and in after 360 days the means exceed those presented in the pre-HSCT stage. Regarding fatigue and pain symptoms, they presented the highest averages in the pancytopenia period, in both groups, with gradual reduction during the treatment. It is noteworthy that the pain symptom was more intense in the allogenic group in this period.

**Figure 2:**
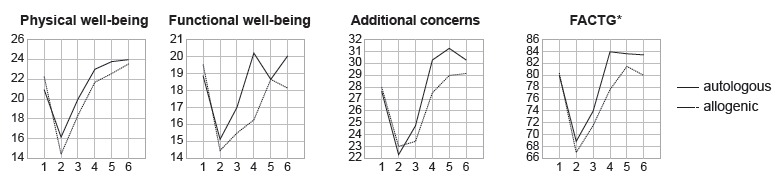
Comparison of the six stages of the research using the domains measured by the Functional Assessment of Cancer Therapy - Bone Marrow Transplantation (FACT-BMT) of patients submitted to autologous and allogenic HSCT. Curitiba, PR, Brazil, 2013-2016, (n=55).

Notes: Friedman test; *FACTG: Overall assessment (physical well-being/social and family well-being/emotional well-being/functional well-being)

Figure 2 shows that, among the six stages of the study, in the domains measured by the FACT-BMT, there was a similar performance between the autologous and allogenic groups between pre-HSCT and the period of pancytopenia. In physical well-being, both groups present gradual recovery of the means between the period of pancytopenia and after 360 days. In functional well-being, the autologous group presented better performance than the allogenic group during the same period. Concerning the domain additional concerns and the FACTG (overall evaluation encompassing physical well-being/social and family welfare/emotional well-being/functional well-being), both groups presented recovery during treatment, however the autologous group presented slightly higher means. 

## Discussion

HSCT is a complex and aggressive treatment, with a large number of variables that must be controlled so that patients’ lives are not jeopardized or their QoL is compromised. During the study, 20 patients (36.36% of the sample) died. The mortality rate associated with treatment is expressed in national and international literature. In Brazil, a study conducted in Campinas-SP, Brazil, with 62 patients submitted to HSCT, showed a death rate of 21% in the period before one year after transplantation^22^. In Iran, a longitudinal study of 587 patients submitted to HSCT revealed that the mean survival time of patients was 517 days^23^.

Despite the significant mortality rate, a significant proportion of patients survives after HSCT and demands support, as well as care at different stages of treatment. In this context, health professionals must know the profile of these patients and the changes they suffer in their QoL during the course of the therapeutic process. 

With regard to sociodemographic analysis, the mean age of the study was 36 years, with extremes between 18 and 69 years. This finding coincides with those found in a study conducted in São Paulo, Brazil, with 71 adult patients submitted to HSCT, whose mean age was 37 years, ranging from 18 to 65 years^24^.

It is important to emphasize that the mean age of the present study belongs to the age group that would normally be at the peak of one’s productive life, inserted in the labor market. Added to this is the fact that 64% of the patients declared themselves to be economically active. This makes treatment an additional concern, since many of them are family providers and, because of their health condition, need to interrupt their professional activities, reducing their family income in a moment of fragility, carrying with them the uncertainty of the future, translated into feelings of fear and anguish. 

In relation to gender, the results of the research showed a small prevalence of males, 53% (n = 29). Corroborating with this result, the statistics of the occurrence of hematological cancer in the Brazilian population in the biannual report 2016-2017 published by INCA estimated that 54% of the cases may occur among men^1^. Men and women suffer from the impact of the diagnosis and the fear of undergoing aggressive and life-threatening treatment. They share the same feelings and concerns about their children or the possibility of infertility. They are uncomfortable with the frailty, both physical and emotional, arising from the treatment and dependence caused by the disease. These factors undoubtedly interfere negatively with their QoL.

In addition, the survey revealed that 55% (n = 30) are married or declared to be in a stable union, data confirmed by similar studies conducted in Brazil and abroad^8^ ^,^ ^25^ ^-^ ^27^. The presence of a spouse or partner can serve as an emotional support to the patient who undergoes transplantation from the time of diagnosis and at different stages of treatment, especially during hospitalization, when social isolation is relatively long.

Regarding the type of transplantation, the results of the present study differ from results found in studies conducted in Spain and India, in which 59% and 70% of HSCTs, respectively, were autologous^28^ ^-^ ^29^. Importantly, the place where the research was carried out is considered a world reference transplantation center also due to the amount of allogenic HSCTs that it performs annually.

The domains of QoL measured by the QLQ-C30 and FACT-BMT instruments made it possible to identify the changes that occur during the first year after the patient undergoes autologous and allogenic HSCT at the different stages of treatment, highlighting the most intense symptoms. The results of this research showed that the overall QOL measured by the QLQ-C30 presented lower mean values in the pancytopenia stage when compared to the pre-HSCT stage; however, there is recovery of these means during the treatment, exceeding the baseline parameters after one year. With the exception of the pre-HSCT stage, in all others, the autologous group presented slightly higher means than the allogenic ones. After one year of HSCT, the means presented above 70 points, indicating a satisfactory performance.

Likewise, the general QoL verified by the FACT-BMT presented means above 90 points in all stages of the research, in both groups, exceeding the pre-HSCT parameters after one year, suggesting that the patients consider their general health status as good. It is emphasized that the gradual recovery of the means throughout the therapeutic process and the exceedance of the baseline parameters demonstrate that the overall QoL improves after one year of HSCT.

These results are corroborated by similar studies conducted at transplantation centers in Germany, Denmark, Finland, Norway, Sweden, Canada, Taiwan and the United States, using QLQ-C30 before and after transplantation, which found that the mean overall QoL becomes reduced during hospitalization; however, they recover at baseline parameter up to one year after transplantation^30^.

A study carried out in Spain evaluated the QoL of patients submitted to autologous and allogenic HSCT and revealed that the overall QoL is worse at two months compared to baseline; however, it improves at nine months after HSCT, confirming a positive evolution throughout the therapeutic process. This leads to conclude that the type of HSCT did not influence the QoL in the evaluated period. Nevertheless, a significant difference between the groups was observed in the physical scale^28^.

The physical function, personal performance and social role domains measured by the QLQ-C30, as well as the physical well-being, functional well-being and additional concerns domains assessed by FACT-BMT, presented lower averages during hospitalization, especially in pancytopenia stage, but there was recovery of baseline values after one year. The comparison of autologous and allogenic groups reveals that the means of the autologous group are slightly higher to the allogenic, but without statistically significant differences. 

The improvement in these domains is an expected result, since in the hospitalization period, mainly in pancytopenia, the patient experiences critical moments of the treatment, when complications can occur that put their life at risk or negatively interfere with their QoL. However, after the hospitalization period, it is expected that they will recover and resume their life and the social life they had before starting treatment. For authors^30^, the pre-HSCT means are reached between seven and 12 months after transplantation.

In the symptom scale, all items presented the highest mean values in the pancytopenia stage, both in the autologous and in the allogenic groups, evidencing a significant increase of the symptoms evaluated in this critical stage of the treatment. In relation to fatigue, there was an increase of more than 25 points in the means of this period when compared with pre-HSCT stage. These means decreased during the first year after HSCT, but remained higher than those at the beginning of treatment, suggesting that this symptom remains, although less pronounced, after one year. It is noteworthy that in the first months the patient suffers from the residual effects of drugs received, since the conditioning phase and many of these have side effects or adverse effects with potential to increase the symptoms, thus impacting the QoL.

As for the loss of appetite symptom, it was more intense in the autologous group only in the pre-HSCT stage; in all other stages, the means of the allogenic group were higher, with a significant difference between the groups after100 days (p = 0.031). National and international studies highlight the loss of appetite as one of the most present symptoms during HSCT; it has a substantial increase during hospitalization and remains a problem for up to six months after HSCT^4^ ^,^ ^8^ ^,^ ^19^ ^,^ ^30^. This symptom must be early detected by the care team in order to implement actions that prevent a possible malnutrition, which may compromise the health and the QoL of this patient.

Another recurrent symptom in patients undergoing HSCT is pain. In this study, the symptom intensified in the pancytopenia stage in both groups, but the allogenic group presented a significant difference (p = 0.004) in relation to the autologous group. This differs from a study carried out in the United States in which there was no significant difference between the types of HSCT^31^. 

Throughout the therapeutic process, some clinical conditions are frequent, such as mucositis, and can provoke this symptom causing discomfort and suffering, thus harming QoL. Pain in HSCT should be early detected and managed, both with pharmacological and non-pharmacological measures. The care team must keep in mind the factors that may be contributing or potentiating this symptom and offer comfort measures, thus contributing to a better QoL. 

A study conducted in Turkey with 82 adult patients submitted to HSCT showed that fatigue, pain and loss of appetite become exacerbated after transplantation and emphasized that patients should be evaluated individually in all domains of QoL, as the results of this evaluation will serve as a basis for nursing to put in place effective interventions that will assist patients in coping with these symptoms^19^. 

The results of the symptoms scale offer indications of changes in the domains and in which stages of treatment these changes occur, enabling the nursing team to carry out a plan of care with actions aimed at the reduction of the symptoms, minimizing the negative effects on QoL.

The general FACTG assessment encompassing the domains of physical well-being, functional well-being, social and family well-being and emotional well-being presented high means, except in the pancytopenia stage, ranging from 74.2 to 84.3 in the autologous group and 71.5 to 81.3 in the allogenic group. These results reflect the good performance and satisfaction of patients during the first year after transplantation. The analysis of the results did not confirm significant differences between the groups. 

The domains of QoL measured in the present study made it possible to identify the changes that occur throughout the therapeutic process. The importance of this evaluation is emphasized by authors^32^, when they state that in order to support these patients, one should look closely at their satisfaction with life through the domains of QoL. They also add that the evaluation of QoL has been increasingly common in the studies, since it allows the professionals to understand and identify patients’ needs more clearly and to approach them comprehensively. The authors^33^ reinforce that changes in questionnaire scores may indicate changes in care needs.

Despite clinical differences, patients submitted to autologous and allogenic HSCT present similar changes in the domains of QoL^34^. This reveals that, despite the fact that HSCT is a rigorous and aggressive treatment that predisposes the patient to a broad spectrum of complications, surviving patients eventually consider their quality of life to be good, gradually regaining their life routine. The nursing team should be as close as possible to the patient, elucidating their doubts, conducting guidelines and also trying to involve the family. They must keep a close eye on the signs and symptoms that impact QoL and try to reverse the picture.

## Conclusion

The diagnosis of hematologic cancer alone is already a stressful factor for the patient and his/her family. Besides this, the treatment to which they will be submitted has numerous risks, including death. The feelings involved are very intense for both the patient and the family members who, in the early stages of treatment, are still shocked due to the load of worries. 

This research made it possible to highlight the changes in the domains of QoL and some symptoms of patients with hematological cancer submitted to HSCT during the first year of treatment. These changes can serve as indicators of the overall satisfaction of patients with their own life, as well as guide the nursing actions in a more specific and individualized manner. 

Among the affected domains measured by QLQ-C30, the symptoms of pain in the pancytopenia stage and loss of appetite after 100 days, both in the group of patients submitted to allogenic HSCT, were statistically significant. Regarding FACT-BMT, the statistical analysis showed a significant result in the functional well-being domain after100 days, in the group of patients submitted to autologous HSCT. These results express changes in the QoL of these patients, revealing impairment in these domains.

Despite the aggressiveness of the treatment, these findings demonstrate that patients undergoing both modalities of HSCT consider their overall quality of life to be relatively good throughout the first year of the therapeutic process. The findings of the present research corroborate studies conducted in Brazil and abroad, serving as indicators on changes in the domains of QoL, since they signal to the care team, especially to nurses, which aspects are bothering the patient and in which stage of the treatment this discomfort occurs. Thus, it allows for more specific and individualized interventions, minimizing impairments in QoL and contributing to a better survival.

However, the lack of both national and international studies on this subject, mainly comparing QoL between autologous and allogenic transplantations, became a limiting condition of this study, since it made it difficult to compare with different results in the literature. However, this research may direct new studies on QoL in oncology, indicating other aspects of the theme.
